# Pediatric Oculo-orbital Tumor Characteristics, Imaging and Histopathology Agreement in a Tertiary Level Teaching Hospital, Ethiopia

**DOI:** 10.4314/ejhs.v32i2.12

**Published:** 2022-03

**Authors:** Semira Abrar Issa, Amal Saleh Nour, Getachew Assefa Neknek

**Affiliations:** 1 Department of Radiology, College of Health Sciences, Addis Ababa University, Addis Ababa, Ethiopia; 2 Department of Radiology, College of Health Sciences, Addis Ababa University, Addis Ababa, Ethiopia; 3 Department of Radiology, College of Health Sciences, Addis Ababa University, Addis Ababa, Ethiopia

**Keywords:** CT, Histopathology, MRI, oculo-orbital, Retinoblastoma

## Abstract

**Background:**

Oculo-orbital tumors are frequently encountered pathologies and late diagnosis results in high morbidity and mortality in developing countries. This study aims to assess the computed tomography and magnetic resonance imaging patterns of pediatric oculo-orbital tumors with particular emphasis on retinoblastoma and compare agreement between imaging and histopathology diagnosis.

**Methods:**

A retrospective analysis of 101 pediatric patients with oculo-orbital lesions from February 2017 to January 2020 at Tikur Anbessa Specialized Hospital oncology center. Medical records were reviewed for clinical data, history, physical examination, pretreatment eye exam under anesthesia (EUA), computed tomography (CT) magnetic resonance imaging (MRI) and histopathology reports. The agreement between imaging and histopathology diagnosis was analyzed.

**Results:**

Malignant oculo-orbital tumors represented 97 (96.1%) cases. Age group 2–5 years had 56 (55.4%) cases of oculo-orbital tumors. Retinoblastoma accounted for 78 (77.2%) followed by rhabdomyosarcoma in 8 (7.9%) patients. The primary patient complaint was proptosis in 78 (77.2%) followed by leukocoria in 16 (15.8%). In 88 (89.7%) cases, there was agreement between imaging and histologic findings with 72 out of the 75 histopathology confirmed cases of retinoblastoma showing an agreement. Retinoblastoma patients presented at an advanced stage with orbital and intracranial extension.

**Conclusions:**

In conclusion, patients with oculo-orbital tumors presented with advanced stage of disease. Excellent imaging and histopathology agreement was demonstrated.

## Introduction

Pediatric oculo-orbital tumors are unique with different clinical and imaging characteristic features peculiar to this age group that demands proper recognition for prompt detection and early management (1).

In developing countries, malignant lesions are the most common cause of pediatric oculo-orbital tumors with most of these representing retinoblastoma (2–4). This is in contradiction to the reports from some of the developed nations with lower percentages of malignant tumors in the pediatric age group within the range of 10–32% (5, 6).

The most common clinical presentation of pediatric patients with primary oculo-orbital tumors is protrusion of the eyeball (proptosis) followed by swelling and reddening of conjunctivae and eyelids which often represent late findings (7, 8).

Oculo-orbital lesions can be primarily intraocular or orbital based on anatomic site of origin. In addition to the primary orbital lesions, there are secondary orbital lesions that originate from structures adjacent to the orbit including the intraocular space and metastatic orbital lesions. Orbital tumors are further classified as :1) primary lesions, that originate from the orbit, 2) secondary lesions, that extend to the orbit from adjacent structures; and 3) metastatic tumors (9).

Orbital lesions are also classified into intraconal and extraconal space depending on their relationship with the muscle cone. The muscle cone is formed by the extraocular muscles of the eye and their intermuscular septae. This is important in narrowing the differential diagnosis (10).

Retinoblastoma is the most common intraocular tumor of the pediatric age group with orbital extension in advanced stages. However, the number of secondary orbital retinoblastoma is significantly reduced in the developed nations (5, 7). Retinoblastoma is usually diagnosed by fundoscopy under general anesthesia and ultrasonography. Pathology remains the gold standard to assess high-risk features of retinoblastoma (11). Imaging assesses the extent of the disease.

CT and MRI have brought about a major advancement in the evaluation of oculo-orbital tumors. Imaging can be used to precisely localize a lesion, to help establish a diagnosis, generate a differential diagnosis that guides management and also to follow a known lesion for progression (10). CT is the modality of choice for evaluation of the bony orbit and for calcifications. MRI on the other hand, with its high tissue contrast resolution is superior for evaluation of the visual pathways, the globe and soft tissue (12).

A study analyzing the accuracy of CT in the diagnosis of orbital tumors shows accurate diagnosis can be made based on contrast enhanced CT of benign lesions with a sensitivity of 90.3% and less accurate in diagnosing malignant tumors with a sensitivity of 78.9%. The overall diagnostic accuracy of CT in diagnosing orbital tumors being 86% (13). Another prospective cross-sectional study conducted, over the period of one year, shows even better sensitivity (around 95%) of CT in diagnosing ocular and orbit lesions (14).

Imaging exams are important tools for tumor staging. Advancement in cross sectional imaging techniques also allows integration of CT and MRI images into radiation planning systems (15). Understanding the most common oculo-orbital lesions in our setup, their typical clinical presentation and imaging appearance allows for the best use of different imaging techniques in reaching the correct diagnosis. Proper utilization of such imaging techniques like CT and MRI tailored to the patients' maximum benefit is also of upmost importance in pediatric patients.

The aim of this study was to assess the sociodemographic characteristics, imaging pattern and histopathologic agreement of pediatric oculoorbital tumors in the Ethiopian setup with particular emphasis given to retinoblastoma.

## Materials and Methods

A retrospective institutional based cross sectional study design was employed to assess the clinical and imaging pattern of oculo-orbital tumors as well as agreement with histopathologic findings. The study was conducted at TASH oncology unit from February 2017 – January 2020. TASH is the largest tertiary referral hospital in the country with the only cancer center that provides radiotherapy.

The source population were all pediatric patients with oculo-orbital tumor who were referred to the oncology department during the study period. The study population were all pediatric patients under the age of 14 with oculoorbital tumor who have both cross-sectional imaging and pathology result being evaluated at the oncology unit during the study period.

Patients who are previously treated, those with early-stage retinoblastoma without EUA as well as patients whose medical records were irretrievable or incomplete were excluded from the study.

The medical records of 134 patients were reviewed, 33 had incomplete data and were excluded leaving 101 patients. The patient's demography, history, physical examination, EUA , CT, MRI reports and histopathology reports were reviewed and filled on a structured questionnaire. For patients with bilateral disease, information was taken for the most severely affected eye.

**Data analysis**: Data entering, coding and clearing for the quantitative data was performed using Microsoft excel and the analysis was performed with SPSS version 23. The socio-demographic and clinical characteristics of participants were computed by using simple descriptive statistics (mean, percentage, frequencies). Means and ranges were calculated from continuous variables. The agreement between imaging and histopathology diagnosis was analyzed using cross tabulation.

Agreement between imaging and histopathology is present when the imaging diagnosis or the first imaging diagnosis in the list of differential diagnosis is similar with the histopathology diagnosis.

**Ethical consideration**: Ethical clearance was obtained from the ethics committee of the department of radiology before the commencement of the study.

## Results

We reviewed 134 medical records of which 101 fulfilled the inclusion criteria; 48 (47.5%) male and 53 (52.5%) female with a male-to-female ratio of 1:1.1. The age group 2–5 years had 56 (55.4%) patients. The primary complaint was protrusion of the eye seen in 78 (77.2%) followed by leukocoria 16 (15.8 %).

Pathology specimen exam was performed from the orbital mass either by excisional biopsy 70 (71.4%) or incisional biopsy 16 (16.3%). Peripheral smear (PS)/bone marrow aspiration (BMA) rendered diagnosis in five (5.1%) of patients. Biopsy/ FNAC from enlarged lymph nodes confirmed the diagnosis in seven (7.1%) of the cases. According to IIRC staging, three patients presented early and had fundoscopic and ultrasound examination to make a diagnosis.

Malignant tumors were seen in 97 (96.1%) with benign lesions representing four (3.9%) of the cases. Primary orbital tumors were seen in 11(17 %) and distant metastasis in 9 (13.8%). Lesions with orbital extension represented 45 (69.2 %) of the cases including the 43 retinoblastoma patients and 2 nasopharyngeal carcinomas.

MRI was used as the imaging modality in 61(60.4%) and CT scan was done for 34 (33.7%) with six (5.9%) of the patients having both CT and MRI scans at initial diagnosis.

Based on the imaging findings, both the intraocular and extraocular space are involved in 45 (44.5%) of the cases followed by isolated intraocular space tumors in 35 (34.7%) and with isolated extraocular space involvement in 21(20.8%) of the cases.

**Figure 1:**
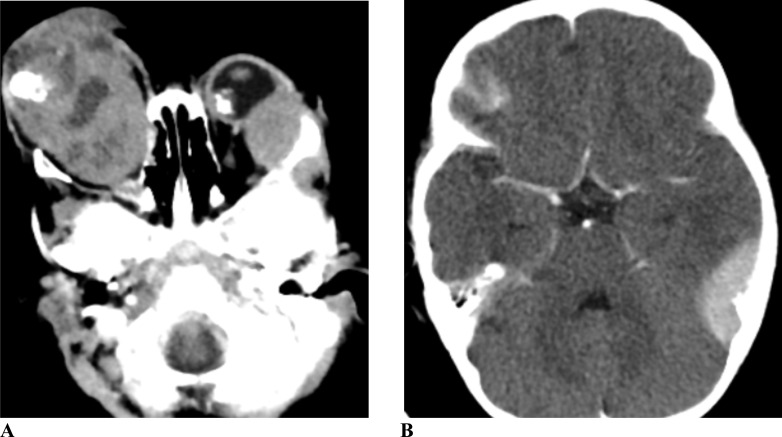
Post-contrast axial brain CT of a 2 year-old female with bilateral retinoblastoma showing heterogeneously enhancing mass replacing the right globe and involving all the subspaces of the orbit having internal areas of dense calcification. There is also focal lesion in the posteromedial wall of the left eye globe. There are homogenously enhancing lesions involving extraconal space in the left orbit (A) and extra-axial space in the left occipitotemporal convexity (B) suggestive of intracranial metastasis.

**Figure 2:**
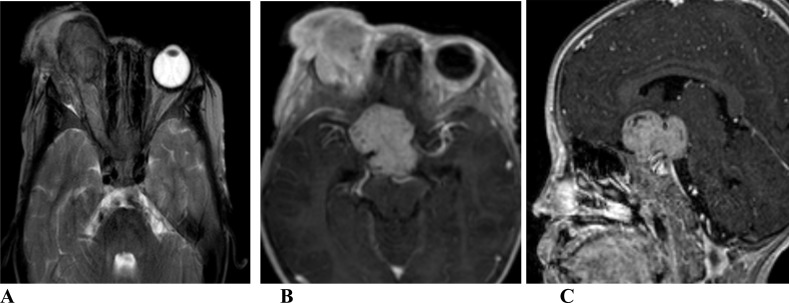
MRI of a 4 years-old female patient presenting with right eye swelling of 2 month duration. There is right intraocular ill-defined mass replacing most of the globe with proptosis. The mass has intermediate signal on T2 W (A) with intense contrast enhancement (B). There are similar signal intensity change following the right optic nerve and optic chiasm forming a suprasellar mass (B, C).

Retinoblastoma represented 78 (77.2%) of the oculo-orbital tumors diagnosed in our study as shown in Table 1. The mean age of retinoblastoma diagnosis was 3.26 years (±20wk). The lesions were unilateral in 59 (75.6%) and bilateral in 19 (24.4%) cases (Figure 1 and 2). Trilateral retinoblastoma was seen in 3 (3.8%) of the cases. There were 23 (29.5%) patients with intracranial extension. This includes, intracranial mass 15 (19.2%), prechiasmatic lesion 5(6.4%) and leptomeningeal involvement in five (6.4%). All patients with intraocular retinoblastoma had eye examination under anesthesia (EUA) for staging. Based on IIRC staging system 32 (91.4%) out of the 35 intraocular retinoblastoma cases were stage E followed by two (5.7%) stage D and one (2.9%) stage C as shown in (Table 2).

**Table 1:** Gender and Age characteristics of pediatric patients diagnosed with orbital tumors at TAH from February 2017–January 2020

Variables		Frequency	Percent
Gender	Male	48	47.5
	Female	53	52.5
Age range(in years)	<1	11	10.9
	1–2	21	20.8
	2–5	56	55.4
	5–10	11	10.9
	>10	2	2.0

**Table 2:** Types of pediatric oculo-orbital tumors in patients evaluated during the period of February 2017–January 2020

Diagnosis	Frequency	Percent
Retinoblastoma	78	77.2
Rhabdomyosarcoma	8	7.9
Optic glioma	2	1.98
Orbital lymphangioma	1	0.99
Nasopharyngeal	2	1.98
carcinoma	1	0.99
Capillary hemangioma	5	4.95
Neuroblastoma	4	3.96
Leukemia		

There were no patients with stage A and Stage B. Based on the IRSS staging system (Table 3), patients were staged as IVb in 23 (29.5%) of the cases and stage IIIa in 22 (28.2%) of the cases. Patients were stage I in 12 (15.4 %) and stage II 12 (15.4%). Stage 0, IIIb and IVa was found in equal proportion each representing three (3.8%) of cases.

**Table 3:** IIRC staging of intraocular pediatric patients with intraocular retinoblastoma evaluated in TAH from February 2017–January 2020

IIRC stages	Frequency(n)	Percent (%)
A	-	-
B	-	-
C	1	2.9
D	2	5.7
E	32	91.4

There were eight patients with rhabdomyosarcoma (RMS), the subtype was mentioned in seven patients from which four had alveolar type of RMS, two embryonal type and one pleomorphic type. In patients with orbital metastasis, the mean age at presentation was 5.3yrs (± 34wk) and 7 (77.8%) of the patients were in the age range of 5 to 10yrs. The origin of orbital metastasis in our series was from neuroblastoma 5 (55.6%) and leukemia 4 (44.4%). From the 98 patients with both imaging and pathologic diagnosis, agreement between imaging and pathologic diagnosis was seen in 88 (89.8%) with disagreement seen in the remaining 10 (10.2%). The agreement for retinoblastoma with histologic diagnosis was in 72 cases out of the 75 (96%).

## Discussion

Among 101 pediatric patients with oculo-orbital tumors included in our study, malignant tumors represented the vast majority in 97 (96.1%) of the cases. The higher percentage of malignant tumor in our study is likely attributed to the study centre being the only referral hospital with radiotherapy unit and most of the patients referred could comprise of malignant lesions (9).

The majority of patients were ≤ 5yrs of age representing 88 (87.1%) of the cases with the mean age at presentation being 3 years and 6 months. This is in line with the studies in an Indian population with most of the paediatric orbital tumours found below age of 6 years (16, 17).

Proptosis was the most common complaint at presentation followed by leukocoria. A study done in Ethiopia also found proptosis as the most common presenting sign which was seen in 53.7% of the cases (18). A Kenyan study reported leukocoria as the primary complaint with lower percentage of proptosis (37%) (19). This shows the large number of patients presenting with late or advanced stage of oculo-orbital tumor in our setup.

The most common oculo-orbital tumor in our study was retinoblastoma representing 78 (77.2%) cases which compares favorably with the study done in Nigeria (2) who reported retinoblastoma as the most common cause of oculo-orbital tumor accounting for 76% of the cases. The mean age of retinoblastoma diagnosis was 38.6 months in our study. This was comparable with a Kenyan reported mean age of 39.9 months. An Ethiopian study reported mean age for the right eye of 34.4 and left eye of 40.2 months.(18, 19)

Bilateral retinoblastoma was found in 24.4% of the cases and unilateral retinoblastoma in the remaining 75.6% of the cases in our series. An Ethiopian study on the presenting signs of retinoblastoma reported bilateral retinoblastoma in 22% of the cases (18). Trilateral retinoblastoma was found in 3 (3.8%) of the cases in our series and all are pineal trilateral retinoblastoma. Potter et-al reported 3% incidence of trilateral retinoblastoma (21). A meta-analysis reported an adjusted incidence of 3.8% with 2.9% pineal and 0.7% non-pineal trilateral retinoblastoma (22).

Based on the IIRC scheme, the majority of the patients in our study are grouped under category E (32,91.4%) because of the advanced stage at presentation. This classification scheme is important to implement for early presenting patients who don't require biopsy as seen in three cases in our study which where stage D (2, 5.7%) and C (1, 2.9%). A study in India reported 64% group E, 15% group B and 15% group D retinoblastoma (23). Based on the IRSS staging system, patients in our study were most commonly extraocular, staged as IVb (23,29.5%) followed by stage IIIa (22,28.2%). The same Indian study reported 27.7% of cases having extraocular extension and from these, the majority (84.3%) were stage III (23). The late diagnosis in our setup with advanced stage at presentation could be from poor awareness on the caregivers/parent's side on identifying early signs such as leukocoria, socioeconomic reasons or delayed referral system. This can be evidenced by research done in Saudi Arabia, in a similar institute over a 20-year time gap, after increased awareness of the society and improved healthcare system resulted in significant decrement in the number of patients presenting with advanced retinoblastoma (7, 24).

For patients with orbital metastasis, the mean age at presentation was 5yrs and 3month which was lower than the one reported by Bidar et al which was 8yrs (25). The most common origin of orbital metastasis was neuroblastoma followed by leukaemia (AML and ALL). Similar findings were seen in the U.S.A and Saudi Arabian studies with neuroblastoma and leukemia as the dominant cause of orbital metastasis in pediatric patients (5, 24).

The agreement rate between imaging and pathologic diagnosis for oculo-orbital tumors in general was 89.7% with disagreement seen in the remaining 10.3% of the cases. There was strong agreement of 98.6% seen for retinoblastoma. Two patients who were diagnosed as retinoblastoma and RMS on imaging but a pathology diagnosis of phthisis bulbi was made. Seema Kashyap and his colleagues reported phtisis bulbi in 3.5% of patients with retinoblastoma with most of the patients having advanced retinoblastoma (26). These two patients had a final diagnosis of retinoblastoma based on clinical findings and examination under anesthesia (EUA). While imaging is a valuable diagnostic tool in the interpretation of orbital tumors, it is most accurate when conducted in the context of the patient's medical history and clinical exam (27).

The imaging and pathologic correlation showed disparity in orbital RMS with 3 out of 8 (37.5%) patients having other diagnosis. Two cases considered hemangioma at initial imaging and one of the patients had an unspecified diagnosis. RMS may sometimes be highly vascular with internal flow voids masquerading as infantile hemangioma. It is important to correlate with the clinical history as RMS tends to present with rapidly progressive proptosis (28).

There was also disparity between imaging and MRI finding for metastasis with 2 out of 9 patients (22.2%) considered as orbital RMS and optic glioma on MRI. These two patients had primary tumor elsewhere and it shows the importance of putting clinical findings into consideration during imaging interpretation of patients with orbital tumors (29). There were small number of patients with orbital RMS and metastasis in our series and further studies are recommended.

The limitation of the study is the lack of case variety due to the set-up of the institution being at the only radiotherapy center the study was conducted. Involving multiple tertiary level institutions might have averted this.

In conclusion, oculo-orbital tumor with advanced stage at presentation with excellent imaging and histopathology agreement was demonstrated. It is the recommendation of this study to create awareness about oculo-orbital tumors as there is a varied appearance as well as degree of malignancy. Identifying early signs and symptoms of oculo-orbital lesions in order for timely detection could prevent advanced stage at presentation.

## Figures and Tables

**Table 4:** IIRS staging of pediatric patients retinoblastoma evaluated in TAH from February 2017–January 2020

IIRS stages	Frequency(n)	Percent (%)
0	3	3.8
I	12	15.4
II	12	15.4
IIIa	22	28.2
IIIb	3	3.8
IVa	3	3.8
IVb	23	29.5
